# Draft Genome Sequences of Rhodotorula mucilaginosa Strains Isolated from the International Space Station

**DOI:** 10.1128/MRA.00570-20

**Published:** 2020-07-30

**Authors:** Robert Daudu, Ceth W. Parker, Nitin K. Singh, Jason M. Wood, Marilyne Debieu, Niamh B. O’Hara, Christopher E. Mason, Kasthuri Venkateswaran

**Affiliations:** a Jet Propulsion Laboratory, California Institute of Technology, Pasadena, California, USA; b Biota, New York, New York, USA; c Department of Cell Biology, College of Medicine, SUNY Downstate Health Sciences University, Brooklyn, New York, USA; d Department of Physiology and Biophysics, Weill Cornell Medicine, New York, New York, USA; e The WorldQuant Initiative for Quantitative Prediction, Weill Cornell Medicine, New York, New York, USA; University of California, Riverside

## Abstract

The whole-genome sequences (WGS) of 28 isolates from the International Space Station were generated and identified as Rhodotorula mucilaginosa, a pigmented yeast that has been classified as an emerging human pathogen in recent times. These WGS enable the identification of genes responsible for synthesizing compounds with biological implications.

## ANNOUNCEMENT

Rhodotorula mucilaginosa of phylum *Basidiomycota* is found in soil, air, food, stool, and other environments (1) and produces carotenoids, making it easily identifiable by its distinctive pink, yellow, orange, or red colonies (2). Carotenoids are important for various biological activities, including vitamin A biosynthesis, enhancement of the immune system, reduction of the risk of various diseases (3), and protection from radiation (4). For these reasons, *R. mucilaginosa* carotenoids are used as food additives and hold pharmaceutical potential (5). *R. mucilaginosa,* which was previously considered to be nonpathogenic, has now been classified as an emerging pathogen (6, 7) and has been shown to colonize central venous catheters, causing fungemia due to biofilm formation (8).

Among the 28 recognized members of the genus *Rhodotorula* (9), *R. mucilaginosa* is the most common species isolated from the environment (7) and the most abundant yeast isolated from surfaces of the International Space Station (ISS) (10). The ability of this yeast to produce biofilms makes it very important to study ISS strains since the harsh conditions of the ISS (microgravity and radiation) were shown to enhance antimicrobial resistance and biofilm formation (11, 12). Due to their ability to form biofilms and colonize life support systems, such as water tanks and pipes containing clean water, characterization of whole-genome sequences (WGS) of *R. mucilaginosa* would allow for the development of countermeasures to eradicate this potential threat.

Samples were collected from ISS surfaces using premoistened polyester wipes (10). Each sample was aseptically transferred into 200 ml of phosphate-buffered saline, vigorously shaken, and concentrated using an InnovaPrep (Drexel, MO) CP-150 concentrated pipette. A 100-μl aliquot from each sample was plated onto potato dextrose agar (PDA) with 100 μg/ml chloramphenicol (25°C; 7 days). A single colony was obtained and restreaked onto PDA plates (25°C; 7 days), and a single colony was collected for DNA extraction. Genomic DNA was extracted by using a ZymoBIOMICS DNA MagBead kit (Zymo, Irvine, CA).

To acquire the WGS of these 28 fungal strains, shotgun libraries were prepared using the Illumina Nextera Flex protocol (13). Paired-end sequencing was performed on a NovaSeq 6000 S4 flowcell paired-end (PE) 2 × 150-bp platform. Quality analysis was performed with FastQC (v0.11.7) (14) to validate the quality of the raw sequencing data. For quality control, adapter trimming and quality filtering were performed using the software fastp (v0.20.0) (15), and then the cleaned sequences were assembled using SPAdes (v3.11.1) (16). Three functions of fastp were used, namely, correction of mismatches in overlapped regions of paired-end reads, trimming of autodetected adapter sequences, and quality trimming at the 5′ and 3′ ends. SPAdes ran using an option to reduce the number of mismatches and short indels in the final contigs, the automatic read coverage cutoff value, and the default values of k-mer sizes. To assess the assembly quality, the number of contigs, *N*_50_ values, median coverage, and the genome size were calculated using QUAST (v5.0.2) (17) (Table 1). The G+C content ranged between 60.53% and 60.55%. All other statistics are given in Table 1.

**TABLE 1:** Genome statistics of Rhodotorula mucilaginosa isolated from various ISS environments during microbial tracking^*a*^

Sample name	GenBank accession no.	Raw sequence accession no.	Flight/location	Location description	No. of contigs	Genome size (bp)	*N*_50_ (bp)	Median coverage (×)	No. of passed filter reads
IF1SW-B1	JABBIR000000000	SRR11774209	F1-1	Cupola (node 3)	177	20,046,905	330,870	129.91	28,317,184
IF1SW-F2	JABBIH000000000	SRR11774205	F1-1	Cupola (node 3)	198	20,124,384	333,776	84.36	19,015,638
IF3SW-F2	JABBIG000000000	SRR11774204	F1-3	ARED (node 3)	201	20,117,457	333,691	97.77	21,673,000
IF4SW-B1	JABBIQ000000000	SRR11774208	F1-4	Dining table (node 1)	187	20,115,049	329,462	140.63	33,186,328
IF4SW-B2	JABBIP000000000	SRR11774197	F1-4	Dining table (node 1)	170	20,047,348	332,671	140.63	30,611,592
IF4SW-F2	JABBIF000000000	SRR11774203	F1-4	Dining table (node 1)	185	20,043,495	330,890	88.39	19,426,356
IF5SW-F1	JABBIE000000000	SRR11774202	F1-5	Zero G stowage rack	192	20,113,158	332,417	139.26	31,068,638
IF6SW-B2	JABBYN000000000	SRR11774188	F1-6	PMM port 1	179	20,045,004	359,523	129.91	28,411,998
IF6SW-F1	JABBID000000000	SRR11774201	F1-6	PMM port 1	180	20,050,344	331,252	136.61	30,528,434
IF7SW-B3	JABBIO000000000	SRR11774187	F1-7	Lab 3 overhead	192	20,045,846	339,159	124.55	26,791,674
IF8SW-B2	JABBIN000000000	SRR11774186	F1-8	Port crew quarters (node 2)	188	20,043,142	352,443	140.63	36,119,534
IF8SW-P2	JABBIM000000000	SRR11774185	F1-8	Port crew quarters (node 2)	192	20,113,185	319,608	135.27	35,567,466
IIF1SW-F1	JABBIC000000000	SRR11774200	F2-1	Cupola (node 3)	203	20,113,961	335,522	93.75	20,465,404
IIF2*SW-B1	JABBII000000000	SRR11774206	F2-2	WHC	184	20,052,772	275,091	113.84	27,351,418
IIF2SW-F1	JABBMW000000000	SRR11774199	F2-2	WHC	180	20,050,420	343,644	140.63	30,717,766
IIF2*SW-F1	JABBIA000000000	SRR11774194	F2-2	WHC	199	20,045,739	311,341	95.09	20,483,810
IIF4SW-F1	JABBMV000000000	SRR11774198	F2-4	Dining table (node 1)	178	19,988,416	334,586	68.30	14,736,170
IIF5SW-F2	JABBMU000000000	SRR11774196	F2-5	Zero G stowage rack	173	19,996,184	340,304	152.68	33,355,092
IIF6SW-B1	JABBMX000000000	SRR11774184	F2-6	PMM port 1	201	20,114,311	317,098	123.21	29,834,278
IIF6SW-B2	JABBIL000000000	SRR11774183	F2-6	PMM port 1	193	20,045,085	311,342	132.59	29,218,554
IIF6SW-F1	JABBYM000000000	SRR11774193	F2-6	PMM port 1	188	20,045,112	294,049	95.09	20,554,260
IIF8SW-B2	JABBIK000000000	SRR11774182	F2-8	Port crew quarters (node 2)	172	20,044,451	330,156	95.09	20,617,330
IIF8SW-B3	JABBIJ000000000	SRR11774207	F2-8	Port crew quarters (node 2)	175	20,050,813	328,275	103.13	22,961,434
IIF8SW-F1	JABBIB000000000	SRR11774195	F2-8	Port crew quarters (node 2)	173	20,047,674	343,393	125.89	27,516,784
IIFCSW-F1	JABBHZ000000000	SRR11774192	F2-FC	Field control wipe	188	20,117,057	331,823	101.79	22,300,810
IFCSG-B1	JABBHY000000000	SRR11774191	Ground CRV-5	Inside capsule CRV5 (FC)	177	20,050,250	321,788	112.5	25,568,744
IF1SG-B1	JABBHX000000000	SRR11774190	Ground CRV-5	Outside capsule CRV5 (L1)	176	20,053,156	335,912	108.48	24,942,204
IF3SG-B1	JABBHW000000000	SRR11774189	Ground CRV-5	Inside capsule CRV5 (L3)	185	20,046,360	317,139	156.70	35,073,840

a Abbreviations: F1 and F2, flight 1 and 2, respectively; ARED, advanced resistive exercise device; WHC, waste and hygiene compartment; PMM, permanent multipurpose module; CRV, crew resupply vehicle; FC, field control.

### Data availability.

This whole-genome shotgun project has been deposited at DDBJ/ENA/GenBank under the accession numbers given in Table 1 (BioProject no. PRJNA625575). The version described in this paper is the first version.
